# Polycystic Ovary Syndrome, Insulin Resistance, and Obesity: Navigating the Pathophysiologic Labyrinth

**DOI:** 10.1155/2014/719050

**Published:** 2014-01-28

**Authors:** Joselyn Rojas, Mervin Chávez, Luis Olivar, Milagros Rojas, Jessenia Morillo, José Mejías, María Calvo, Valmore Bermúdez

**Affiliations:** ^1^Endocrine and Metabolic Diseases Research Center, School of Medicine, University of Zulia, Maracaibo 4004, Venezuela; ^2^Institute of Clinical Immunology, The University of Los Andes, Mérida 5101, Venezuela

## Abstract

Polycystic ovary syndrome (PCOS) is a highly prevalent endocrine-metabolic disorder that implies various severe consequences to female health, including alarming rates of infertility. Although its exact etiology remains elusive, it is known to feature several hormonal disturbances, including hyperandrogenemia, insulin resistance (IR), and hyperinsulinemia. Insulin appears to disrupt all components of the hypothalamus-hypophysis-ovary axis, and ovarian tissue insulin resistance results in impaired metabolic signaling but intact mitogenic and steroidogenic activity, favoring hyperandrogenemia, which appears to be the main culprit of the clinical picture in PCOS. In turn, androgens may lead back to IR by increasing levels of free fatty acids and modifying muscle tissue composition and functionality, perpetuating this IR-hyperinsulinemia-hyperandrogenemia cycle. Nonobese women with PCOS showcase several differential features, with unique biochemical and hormonal profiles. Nevertheless, lean and obese patients have chronic inflammation mediating the long term cardiometabolic complications and comorbidities observed in women with PCOS, including dyslipidemia, metabolic syndrome, type 2 diabetes mellitus, and cardiovascular disease. Given these severe implications, it is important to thoroughly understand the pathophysiologic interconnections underlying PCOS, in order to provide superior therapeutic strategies and warrant improved quality of life to women with this syndrome.

## 1. Introduction

Polycystic ovary syndrome (PCOS) is an endocrine-metabolic disorder characterized by multiple hormonal imbalances, reflecting on a clinical presentation dominated by manifestations of hyperandrogenism, which generate short and long term consequences on female health [1]. Among these, infertility is one of the most alarming associated morbidities, as it currently affects approximately 48.5 million women aged 20–44 years [2], with PCOS accounting for 6–15% of these cases [3], although up to 70% of women with PCOS may be undiagnosed [4]. Indeed, its optimal diagnosis is often hindered due to its apparent similarities with several other pathologies remarkably, obesity as well as Cushing's syndrome, ovarian and adrenal neoplasms, and congenital adrenal hyperplasia [5].

The manifestations of PCOS are not confined to the gynecological sphere; women afflicted by this disease show an increased prevalence of several comorbidities, including obesity, dyslipidemia, hypertension, metabolic syndrome (MS), and type 2 diabetes mellitus (DM2) in comparison with women without PCOS. These features, along with other alterations such as endothelial dysfunction and a chronic low-grade inflammatory state, underlie the greater risk of developing cardiovascular disease and increased all-cause mortality observed in these subjects [6].

Amongst the complications previously mentioned, obesity stands out as it has reached epidemic proportions [7], with a worldwide prevalence of 35% in females and up to 55% in South America and the Caribbean [8]. Furthermore, in our locality, 32.4% of women are obese [9]. Indeed, both PCOS and obesity boast highly concerning prevalence figures [10]. Several studies have pinpointed insulin resistance (IR) as the fundamental link associating these conditions [11], although IR may be present in PCOS independently of obesity [12]. IR, defined as a metabolic state characterized by a decrease in cellular ability to respond to insulin signaling, appears to be an essential pathophysiologic mechanism in the development of all metabolic complications of PCOS [13]. As a consequence, outstanding proportions of women with PCOS are also diagnosed with DM2 or MS, as well as isolated criteria from the latter [14]. Compensatory hyperinsulinemia appears to mediate many of these deleterious effects. This phenomenon stems as a response of pancreatic *β* cells in order to preserve lipid and carbohydrate homeostasis in face of diminished insulin sensitivity [15]. This compensation leads to *β* cell exhaustion and the genesis of not only DM2, but also a series of collateral effects originated by hyperinsulinemia, including the aforementioned frequent comorbidities of PCOS [16].

Notwithstanding the importance of IR in the development of PCOS, both obese and nonobese patients have specific mechanisms leading to ovarian dysfunction independent of IR, reflecting the complexity of this syndrome [17]. Therefore, considering the severe consequences PCOS exerts on the health and lifestyle of the affected women, it is of utmost importance to unravel the intricate pathophysiologic cross-talk among PCOS, IR, and obesity.

## 2. The Ovarian Cycle in Polycystic Ovary Syndrome: When It All Goes Wrong

Because no specific sole cause for PCOS has been determined, the most accepted premise is a multifactorial model, where interactions between environmental cues and factors intrinsic to each individual act in consonance toward a common result, which is the development of hyperandrogenemia, a biochemical hallmark of this pathology. This alteration is the main culprit behind most clinical manifestations of PCOS [18].

In PCOS, several of the physiological events within the ovarian cycle and folliculogenesis are disrupted. The very beginning of folliculogenesis is compromised due to high levels of Anti-Müllerian Hormone (AMH) [19]. AMH is a 560 amino acid peptide of the TGF-*β* family, which is secreted by granulosa cells (GC) and displays its greatest expression in small antral follicles and exerts powerful inhibition of primordial follicle initiation and follicle sensitivity to follicle-stimulating hormone (FSH). AMH levels progressively decrease as follicles increase in size, and low levels of this hormone appear to be a requirement for transition from the primordial to the primary stage, dominant follicle selection, and progression to ovulation [20]. In women with PCOS, elevated levels of AMH appear to play an important role in long term disruption of ovarian physiology [21], with greater AMH concentrations being linked to worse fertility outcomes [22].

Feedback disturbances in the hypothalamus-hypophysis-ovary axis (HHOA) are another typical feature of PCOS [23], with increased frequency and amplitude of gonadotropin-releasing hormone (GnRH) and luteinizing hormone (LH) pulsatile secretion. Higher levels of this hormone induce greater androgen synthesis in ovarian theca cells (TC) [24]. In turn, hyperandrogenemia induces a decrease in feedback sensitivity to both estradiol and progesterone in gonadotropic hypothalamic cells, reinforcing GnRH and LH hypersecretion [25]. This represents the first of many self-perpetuating pathophysiologic cycles in which hyperandrogenemia plays a pivotal role in the development and progression of PCOS, while simultaneously warranting the presence of the clinical manifestations. The constant growth of follicles, along with nonselection of a dominant unit, leads to the hyperstimulation of several of these structures, hence the alternative proposed denomination of “polyfollicular ovary syndrome” [26], which maintains all the characteristic hormonal imbalances.

Genetic factors are also considered to play an important role in the development of this syndrome, by setting the stage for abnormally high androgen synthesis in ovarian tissue. The most accepted model proposes a probable Mendelian pattern of inheritance, where key genetic defects would be transmitted to offspring in a dominant autosomal fashion, albeit with widely variable penetrance, dependent on several environmental and epigenetic factors, such as *in utero* exposure to elevated levels of androgens [27]. Notoriously, mutations in the genes of the androgen receptor, sex hormone binding globulin (SHBG), and steroidogenic enzymes may be especially important in predisposing to the development of hyperandrogenemia [28].

## 3. Polycystic Ovary Syndrome: The Clinical Picture

In the clinical setting, PCOS presentation is greatly heterogeneous, with a broad clinical manifestation spectrum (Table 1). Several sets of diagnostic criteria for PCOS are presented in Table 2 [29]. Although the presence of oligomenorrhea indicates ovulatory dysfunction, apparent eumenorrhea does not completely rule out anovulation. To this end, progesterone levels <3-4 ng/mL in days 20–24 of the menstrual cycle are sufficient to diagnose the cycle in question as oligo/anovulatory. In contrast, a patient can be diagnosed as anovulatory after ascertaining anovulation in at least 2 subsequent cycles, in the presence of hypoprogesteronemia [30].

Although IR is not traditionally considered a diagnostic criterion of PCOS, the presence of this alteration or *acanthosis nigricans* accompanied by hyperandrogenic signs is highly suggestive of this syndrome [31]. In women with PCOS, obesity is also a very prevalent condition, along with IR. Nevertheless, these may not necessarily be concomitant in all cases, and they may be present independently, translating into distinct metabolic profiles. Each of these phenotypes displays specific biochemical features that render differential profiles, both for cardiovascular risk and fertility outcomes [32].

Because skin is a major target for androgen activity, several hyperandrogenemia-triggered dermatologic alterations can be seen in PCOS, most commonly hirsutism, androgenic alopecia, and acne and also seborrhea, onycholysis, and onychorrhexis [33].

### 3.1. Hirsutism

Hair development is thoroughly affected by hyperandrogenemia. Normally, in females past pubarche the major androgenic molecules are dehydroepiandrosterone sulfate (DHEAS), androstenedione, dehydroepiandrostenedione, testosterone, and dihydrotestosterone (DHT), in descending order of serum concentration. Only the latter two can bind to the androgen receptor and promote hair follicle changes [48, 49]. Hirsutism is defined as the presence of excessive terminal hair in areas of the body that are androgen-dependent and usually hairless or with limited hair growth, such as the face, chest, areolas, abdomen, and upper thighs [50]. Terminal hair should be differentiated from vellus hair, whereas the latter is a longer version of lanugo (the hair that covers fetuses and is shed postnatally) and covers all body surfaces but the lips, palms, and soles; terminal hair is the pigmented, longer, coarser hair that covers the pubic and axillary areas, scalp, eyelashes, eyebrows, and male body and facial hair [48]. Terminal hair development requires androgen stimulation—as seen in pubarche, where androgens trigger vellus to mature into terminal hair [51]—and thus, hirsutism can be seen as the result of the interaction hyperandrogenemia and its influence in the hair follicle unit [52]. It should be differentiated from hypertrichosis [53], which is the overgrowth of vellus in a nonsexual pattern distribution, usually related to persistence of the highly mitotic anagen phase of the hair cycle [54].

Although hyperandrogenemia is the trigger for hirsutism, the rate of hair growth is not proportional to the degree of hyperandrogenism [52], supporting a parallel role for androgen receptor localization (keratinocytes, sebaceous glands, and hair dermal papilla cells) and sensitivity in the development of hair patterns and other skin manifestations, such as acne, alopecia, or seborrhea. Hirsutism can be assessed through the Ferriman-Gallwey Score [55], which evaluates the presence of terminal hair in the upper lip, chin, chest, upper and lower back, upper and lower abdomen, thighs, and arms. Integrated scores from all body areas beyond 15 points are related to a hirsutism diagnosis, although current recommendations suggest the use of the 95th percentile of the score in specific populations, adapting to autochthonous ethnic, hair pattern, and age-related features, in order to diagnose hirsutism properly [52]. Due to the high prevalence of PCOS, all cases of hirsutism during puberty or in women in reproductive age merit determination of sex hormone serum concentrations, as well as pelvic ultrasonography, in order to rule out this syndrome [30].

### 3.2. Acne and Seborrhea

Sebaceous glands are also androgen-dependent structures, with sebocytes being highly sensitive to androgen signaling, which is exacerbated in PCOS, leading to the development of acne and seborrhea [56]. Androgens stimulate sebocyte proliferation—especially in the mid-back, forehead, and chin—and secretion of sebum, a mixture of lipids including glycerides, squalene, free fatty acids (FFA), and cholesterol [57]. Local bacteria further complicate the process by secreting lypolytic enzymes which break down triglycerides produced in the sebocyte. The resulting FFA that are released into sebaceous ducts by apocrine glands are responsible for the characteristic odor observed in these patients [58].

### 3.3. Androgenic Alopecia

On the other hand, androgenic alopecia is a disorder in which hair is miniaturized, due to an increased telogen : anagen ratio—with telogen hair being at mitotical rest and anagen hair being mitotically active—and associated to genetic susceptibility related to increased 5*α*-reductase activity in the hair follicle. This increased enzymatic activity would favor the local conversion of testosterone into DHT, a more powerful androgen [59]. As reported by Cela et al. [60] 67% of the women with alopecia areata have PCOS and elevated levels of testosterone and androstenedione. The balding pattern is dominated by the frontal and parietal scalp zones, leaving the occipital area with great hair density, as opposed to thinner and scarcer hair in the crown area. A variation of this disorder features neither bald spots nor hair loss *per se*, but only shortening in its length and width, manifesting as wispy distal ends [59].

### 3.4. Onycholysis and Onychorrhexis

Finally, nails are also subject to possible alterations in PCOS, in the form of onycholysis—separation of the nail plate from the nail bed due to disruption of the onychocorneal band [61]—and onychorrhexis, splitting of nails in lengthway bridges [62]. The association of these nail conditions with excess androgen is not fully understood, but their presence has been observed to be exacerbated when coexisting with hypothyroidism or dysglycemia [63].

## 4. Insulin Resistance, Hyperinsulinemia, and Hyperandrogenemia: A Vicious Cycle

The role of IR and hyperinsulinemia in the development of PCOS has been thoroughly explored, and it is generally accepted to play an important role in the molecular mechanisms implicated in the androgenic hypersecretion typical of this pathology [64]. This has been evidenced by the decrease in fasting insulin levels observed in women with PCOS that undergo insulin-sensitizing pharmacotherapy, which appears to concurrently lower androgenemia and improve ovarian functionalism [65].

**Figure 1:**
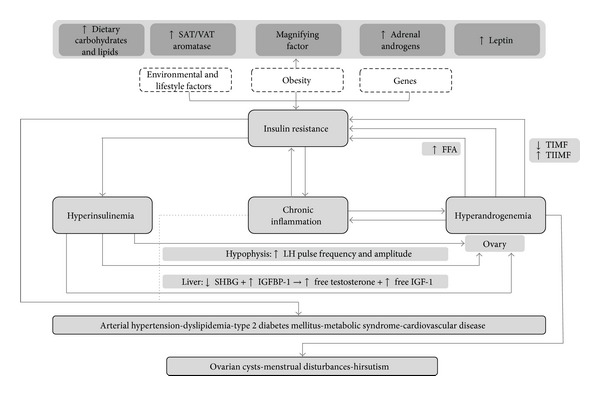
Interactions among insulin resistance, hyperinsulinemia, and hyperandrogenemia in the etiopathogenesis and progression of polycystic ovary syndrome and related comorbidities. SAT = subcutaneous adipose tissue; VAT = visceral adipose tissue LH = luteinizing hormone; SHBG = sex hormone binding globulin; IGFBP-1 = insulin-like growth factor binding protein-1; IGF-1 = insulin-like growth factor-1; FFA = free fatty acids; TIMF = type I muscle fibers; TIIMF = type II muscle fibers. Although details of the exact etiopathogenesis of PCOS are not clear, it appears to be a combination of environmental and genetic factors, which in consonance favor the development of IR. In turn, IR leads to compensatory Hyperinsulinemia, which substantially augments ovarian androgen synthesis by increasing LH pulse frequency at the hypophysis by stimulating GnRH gene transcription in hypothalamic cells. Insulin also triggers hyperandrogenemia by directly activating mitogenic pathways in ovarian cells and increasing transcription of StAR and several key steroidogenic enzymes. Hyperandrogenemia is the key disruption underlying the typical clinical features of PCOS. In addition, increased ovarian production of androgens may in turn worsen IR, thus perpetuating a vicious cycle of IR-hyperinsulinemia-hyperandrogenemia. Indeed, androgens may not only interfere with insulin signaling directly, but also trigger lipolysis, increasing FFA in circulation, favoring IR. Moreover, androgens seem to diminish highly oxidative and insulin-sensitive TIMF and increase glycolytic and less insulin-sensitive TIIMF, further favoring the development of IR. Additionally, obesity appears to magnify all events in this cycle, by increasing androgen synthesis not only in ovaries, but also in SAT and adrenal glands. Leptin also participates by disrupting ovarian physiology and favoring a chronic systemic inflammatory state. Ultimately, both IR and chronic inflammation thrive on all endocrine-metabolic disturbances pertaining PCOS, predisposing patients to the development of comorbidities, such as type 2 diabetes mellitus and cardiovascular disease.

On the other hand, although this association is usually conceived as a one-way relationship from IR to hyperandrogenemia, pathways through which hyperandrogenemia may perpetuate IR and hyperinsulinemia are currently being proposed. Indeed, in the context of PCOS, IR and hyperandrogenemia may assemble a vicious cycle, continuously stimulating each other in a reciprocal fashion. Moreover, this conjunction of endocrine-metabolic alterations sets the stage for the progressive development of additional comorbidities, both metabolic and cardiovascular, further complicating the management of these patients [66]. The main mechanisms suggested within this complex network of interactions are presented in the following paragraphs (Figure 1).

## 5. From Hyperinsulinemia to Hyperandrogenemia: Systematically Disrupting Ovarian Physiology

### 5.1. Insulin and Dysregulation of Hypothalamus-Hypophysis-Ovary and Adrenal Signaling

Insulin may play a part in the development of the typical increased amplitude and frequency of GnRH and LH pulse secretion seen in PCOS. Indeed, elevation of LH and GnRH secretion in response to insulin infusion has been observed *in vitro*, both in dose-dependent and time-dependent fashions [67, 68]. This effect may be mediated by insulin in GnRH-secreting cells of the hypothalamus, by potentiating GnRH gene transcription through the MAPK pathway. As a result, increased GnRH synthesis and secretion lead to a subsequent elevation of LH levels. This continuous stimulation would translate into augmented synthesis of ovarian steroid hormones, particularly androgens [69].

On the other hand, insulin also reinforces adrenal glands as an alternate androgen source parallel to ovaries, by potentiating hypothalamus-hypophysis-adrenal axis (HHAA) activity at several key sites. The hippocampus is an important mediator of HHAA negative feedback, by inhibiting hippocampal activity; insulin indirectly enhances hypothalamic CRH secretion [70] although it may also play a direct role in both the hypothalamus [71] and the hypophysis [72]. Lastly, although the mechanisms remain unclear, insulin appears to augment adrenal cortex sensitivity to ACTH stimulation, with increased androgen secretion [73].

### 5.2. Insulin and Sex Hormone Binding Globulin

Elevated insulin concentrations have been associated with lower levels of SHBG, leading to enhanced bioavailability of androgens [74]. Although insulin and the insulin-like growth factor 1 (IGF-1) have been demonstrated unable to directly repress *shbg* [75], they may be key indirect mediators, as they have been associated with decreased total protein secretion in human hepatic cells [76]. Inhibition of SHBG by elevated concentrations of glucose and fructose is also an important component, mediated by downregulation of hepatocyte nuclear factor 4-*α* (HNF-4*α*) activity. Nonetheless, in the context of IR, this is yet another indirect effect, as it relies on high concentrations of these monosaccharides due to dysfunctional insulin signaling and not on insulin activity *per se* [75]. On the other hand, insulin has been shown to repress insulin-like growth factor-1 binding protein (IGFBP-1) synthesis in a direct, rapid, and complete way in both the liver and the ovaries, allowing for greater IGF-1 availability, which in turn boosts insulin activity not only in the liver—further contributing to lower SHBG levels—but also in the ovaries, reinforcing PCOS pathophysiology [77]. This suppression is mediated by intranuclear thymine-rich insulin response elements (TIRE). Although not all components of the signaling cascade linking the insulin receptor (INSR) with TIRE are currently known, inhibition of GSK-3 through the PI3K pathway appears to be essential in this process [78].

### 5.3. Insulin Signaling in Ovarian Tissue and Selective Insulin Resistance

**Figure 2:**
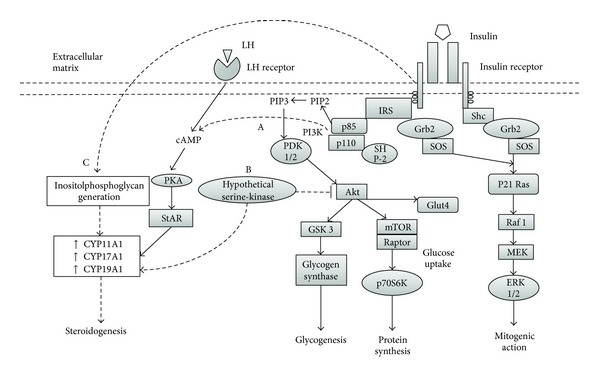
Insulin signaling in ovarian thecal cells and proposed mechanisms for selective insulin resistance. Solid lines represent well-characterized mechanisms. Dashed lines represent incomplete information or hypothetical mechanisms. (A) Insulin may act in synergy with LH to increase intracellular concentration of cAMP, potentiating PKA activation and subsequent phosphorylation of StAR, favoring steroidogenesis. (B) A hypothetical serine-kinase could unify hyperinsulinemia-hyperandrogenemia in PCOS, as it would inhibit Akt activity, negating the metabolic effects of insulin, while activating steroidogenic enzymes. Furthermore, the mitogenic pathway would be left intact and fully functional, favoring thecal proliferation. (C) Inositolphosphoglycan generation appears to be triggered by INSR-prompted mechanisms independent of signaling molecules within metabolic or mitogenic pathways on insulin signaling. Therefore, inositolphosphoglycans may stimulate steroidogenic activity regardless of metabolic and signaling disturbances typical of systemic insulin resistance.

Pleiotropy is a distinguishing feature of insulin signaling, being involved in a wide catalogue of physiologic and pathophysiologic roles through distinct, yet interconnected, second-messenger pathways (Figure 2). For example, phosphorylation of IRS allows it to act as a docking site for other signaling proteins, such as Grb2, NcK, and PI3K, which are crucial for translocation of GLUT-4. Likewise, further downstream in the PI3K pathway is Akt, which mediates activation of GSK, essential for glycogenesis, as well as mTOR, an important step for insulin-induced protein synthesis. A mitogenic pathway is also activated by insulin, through the binding of phosphorylated IRS or Shc with Grb-2/SOS complexes, leading to MAPK activation through p21Ras and Raf-1 [79].

The presence of INSR and IGF-1 receptors in TC, GC, and stromal cells of ovarian tissue unequivocally identifies this organ as a target of insulin activity [80], confirmed by observations of decreased steroidogenesis in TC and GC from both healthy and polycystic ovaries, following *in vitro *administration of both anti-IGF1R and anti-INSR antibodies [81]. One of the key links in this activity is the acute steroidogenic regulatory protein (StAR), a molecule implicated in transportation of cholesterol to the internal mitochondrial membrane, where the cholesterol side-chain cleavage enzyme (CYP11A1) is anchored, the rate-limiting enzyme in steroid hormone synthesis [82]. Insulin appears to augment not only StAR expression, but also CYP11A1, 17-*α*-hydroxylase/17,20-lyase (CYP17A1), 3-*β*-hydroxysteroid dehydrogenase (3-*β*-HSD), and aromatase (CYP19A1) expression, contributing to an excess in the production of progesterone, 17-*α*-hydroxyprogesterone, and testosterone in polycystic ovaries in comparison to healthy ovaries [83].

The central paradox in the pathophysiologic association between hyperinsulinemia and hyperandrogenemia in PCOS is that the ovary remains sensitive to insulin activity and subsequent androgen production, despite a systemic state of IR, setting the stage for the *“selective insulin resistance”* theory [84]. Several mechanisms have been proposed to explain this phenomenon, albeit the true chain of events remains elusive.

#### 5.3.1. cAMP-Dependent Activation of PKA

Insulin appears to act in synergy with LH to elevate intracellular concentration of cAMP, which activates StAR, potentiating steroidogenic activity. Although this effect may be direct through the PI3K pathway, the requirement of cAMP for this activity suggests divergence from the usual insulin cascade; yet the differential molecular interactions are unknown [85]. Similarly, insulin and LH may also act in synergy to increase transcription of LDL-C receptors in GC through the PKA, PI3K, and MAPK pathways. On the other hand, insulin may also augment steroid synthesis through aromatase upregulation in GC, which would serve as substrates for TC for further conversion into androgens [86].

#### 5.3.2. Serine Phosphorylation Theory

This unifying proposal for hyperinsulinemia-hyperandrogenemia in PCOS stems from observations of Dunaif et al., who ascertained a significant decrease in tyrosine-kinase activity, accompanied by considerably higher serine-kinase activity, in fibroblasts of women with PCOS. This differential behavior resulted in attenuated metabolic effects of insulin with normal mitogenesis [87]. Furthermore, in TC, increased serine phosphorylation results in activation of CYP17A1, reflecting into augmented androgen production [88]. Nonetheless, no specific kinase has been evidenced to display these dual effects. However, a key link in the activity of this hypothetical serine-kinase may be the PI3K/Akt pathway, which would explain the coexistence of elevated steroidogenesis with fully functional mitogenic signaling [89].

#### 5.3.3. Inositolphosphoglycan Signaling

Lastly, this pathway stands apart from previous mechanisms as it appears to be independent of all insulin signaling-related molecules except INSR itself [90]. Although the full succession of this cascade is largely unknown, inositolphosphoglycans seem to be able to potentiate steroidogenic activity by stimulating CYP11A1, CYP17A1, and CYP19A1 activity in TC. These observations are noteworthy, as this transduction system may remain intact in the context of IR, in terms of defective tyrosine-kinase activity of INSR substrates and dysfunctional glucose metabolism, warranting ovarian androgen synthesis despite these abnormalities [91].

## 6. From Hyperandrogenemia Back to Hyperinsulinemia: Completing the Loop

Traditionally, the relationship between IR and PCOS is considered to be a unidirectional pathway toward ovarian disturbances. Nonetheless, recent evidence underpins a complex reciprocal interaction between these phenomena. Indeed, in the context of PCOS, hyperandrogenemia *per se* may affect insulin sensitivity [66]. This may be mediated by upregulation of *β*3 adrenergic receptors and hormone-sensitive lipase expression in visceral adipose tissue (VAT) through testosterone or DHEAS signaling [92], modifying lipolytic activity and favoring release of FFA into circulation. This increase in FFA availability causes functional and structural changes in hepatocytes and skeletal myocytes, with the accumulation of metabolites from the long-chain FFA reesterification pathway, including Acyl-CoA and diacylglycerol. In turn, these molecules can activate PKC, a serine/threonine kinase which is widely accepted as pivotal for the mechanisms underlying IR, particularly through serine phosphorylation of IRS-1 [93].

In PCOS, androgens also appear to modify metabolic architecture and functionality in skeletal muscle, by decreasing the amount of type I muscle fibers, which are highly oxidative and insulin-sensitive, and increasing type II fibers, which are glycolytic and less sensitive, as well as decreasing expression of glycogen synthase [94].

Further mechanisms remain poorly characterized, including androgen-driven proinflammatory cytokine secretion from VAT and androgen-induced interference of insulin signaling. In adipocytes, testosterone appears to induce serine phosphorylation of IRS-1 [95], which reflects on inhibition of the metabolic effects of insulin accompanied by normal mitogenic signaling, suggesting that, in PCOS, selective insulin resistance may not only be limited to ovarian tissue, but also be present in adipocytes [96].

## 7. Obesity: A Key Magnifying Factor of Polycystic Ovary Syndrome

### 7.1. Obesity As an IR-Independent Pathophysiologic Factor in PCOS

**Figure 3:**
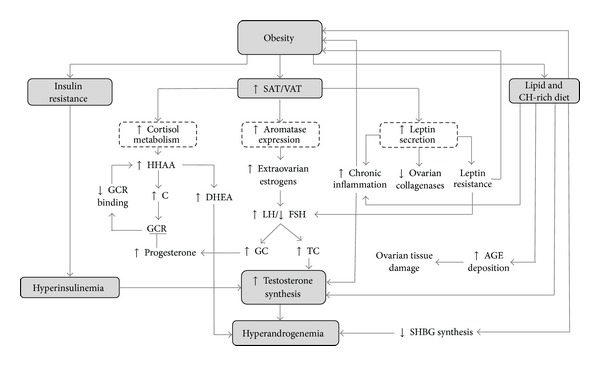
Mechanisms for obesity-mediated potentiation of hyperandrogenemia in polycystic ovary syndrome. SAT = subcutaneous adipose tissue; VAT = visceral adipose tissue; HHAA = hypothalamus-hypophysis-adrenal-axis; C = cortisol; GCR = glucocorticoid receptor; DHEA = dehydroepiandrosterone; LH = luteinizing hormone; FSH = follicle-stimulating hormone; GC = granulosa cells; TC = teca cells; Ch = carbohydrate; AGE = advanced glycation end products; SHBG = sex hormone binding globulin. In PCOS, obesity may potentiate hyperandrogenemia through several mechanisms. Aside from hyperinsulinemia-mediated effects of IR, increased adiposity leads to faster cortisol metabolism, which results in hyperactivation of the HHAA and thus increased DHEA synthesis. Competitive binding to GCR by GC-produced progesterone derives into diminished cortisol binding to GCR, reinforcing HHAA activation. High adiposity also leads to greater aromatase expression in adipocytes, which allows for increased extraovarian estrogen synthesis. In turn, this causes an elevated LH/FSH ratio, prompting hyperplasia of GC and TC, which then synthesizes greater amounts of progesterone and testosterone, respectively. Hyperleptinemia and leptin resistance result in alterations of LH secretion, as well as downregulation of ovarian collagenases, and promotion of a chronic inflammatory state. Carbohydrate-rich diets may induce oxidative stress in circulating mononuclear blood cells and thus chronic inflammation. Lipid-rich diets have been reported to increase testosterone synthesis and downregulate SHBG synthesis, resulting in hyperandrogenemia. These diets also favor endogenous and exogenous AGE deposition, which results in overall ovarian tissue damage.

Menstrual irregularities and anovulation appear to be more prevalent and severe in obese women with PCOS than in their nonobese counterparts, and weight loss of at least 5% tends to be associated with improvement of these conditions. Furthermore, obese women with PCOS have greater long term difficulty for conception [97]. Nevertheless, despite the close association between IR and obesity [98], the latter neither requires IR to influence aspects of PCOS pathophysiology, nor is a *sine qua non* of quality for this entity [99]. Indeed, obesity is a powerful magnifying factor of several aspects of PCOS (Figure 3), which are not limited to favoring the development of IR and hyperinsulinemia.

### 7.2. Obesogenic Dietary Patterns and Hyperandrogenemia

Both short and long term high-lipid, low-fiber diets have been associated with hyperandrogenemia, possibly acting through intake-induced hyperinsulinemia, which would lower SHBG synthesis, increasing androgen availability [100]. Nevertheless, novel mechanisms suggest a direct effect of diet in ovarian physiology disruption. Advanced glycation end products (AGE) are cytotoxic metabolites derived from disrupted carbohydrate metabolism, which may also be exogenously obtained from a myriad of food typical of Westernized diets [101]. AGE deposition in ovarian tissue induces oxidative stress and aberrant structure modification due to molecule cross-linking, leading to damage of all ovarian cell types and therefore altering all aspects of its functionality. Moreover hyperandrogenemia appears to inhibit glyoxalase-I activity, which is an important enzymatic scavenging system for 2-oxoaldehydes, including major precursors of AGE. Thus, in PCOS, the deleterious effects of AGE deposition may be exacerbated [102].

### 7.3. Effects of Obesity on Steroid Hormone Physiology and Metabolism

Aside from disturbances in insulin physiology, obesity implies thorough alterations in steroid hormone metabolism, essentially summarized as increased concentrations of nearly all of these messengers. To this end, hyperestrogenemia is a paramount alteration, stemming from extraovarian estrogen production in VAT and subcutaneous adipose tissue (SAT), due to expression of aromatase in these adipocytes [103]. Estrogens stimulate LH and inhibit FSH secretion, contributing to GC and TC hyperplasia. In turn, this would increase androgen synthesis, which not only cause the typical manifestations of PCOS, but also serve as substrates for extraovarian aromatization, reinforcing this cycle of hyperestrogenemia-hyperandrogenemia in obese women with this pathology [104].

Another common finding in women with PCOS is elevated serum concentrations of adrenal androgens, which suggest dysregulation of the HHAA. Parallel to the effects of insulin in this axis [73], adrenal hyperandrogenemia may be reinforced by increased amounts of VAT, which displays a high traffic and catabolism of cortisol, triggering a compensatory activation of the HHAA, which results in elevation of adrenal androgen levels. Additionally, progesterone may interact with the glucocorticoid receptor, impairing activity of these hormones. As a consequence, hyperestrogenemia, such as that found in PCOS and the luteal phase of the menstrual cycle, may exacerbate this HHAA compensation, leading to higher adrenal androgen production [105], which can also be converted back to estrone in adipose tissue and then into estradiol by 17-*β*-HSD in extraovarian tissues, restarting the hyperestrogenemia-hyperandrogenemia cycle [106]. Moreover, glucocorticoids, which are elevated due to HHAA hyperactivity, induce expression of aromatase, further fueling this positive feedback circuit [103].

### 7.4. The Role of Leptin in PCOS with Obesity

Leptin, known as the prototypical adipokine, is a 167 amino acid peptide secreted primarily from white adipose tissue, although it is present at several other sites, including the ovary [107]. Leptin secretion occurs predominantly in SAT over VAT in women and appears to be inversely related to adipocyte size [108]. Leptin can be found circulating freely—its metabolically active form—or bound to soluble leptin receptor (sOB-R), a carrier protein derived from alternative splicing of leptin receptor (OB-R) mRNA or ectodomain shedding of OB-R transmembrane structures [109]. sOB-R modulates leptin activity by lengthening clearance and half-life, yet limiting its availability to membrane OB-R [110]. Leptin participates in regulation of energy homeostasis and multiple neuroendocrine, immune, and reproductive functions [111].

Indeed, leptin appears to play a permissive role for adequate functioning of the HHOA, as observed in subjects with congenital leptin deficiency, who are typically infertile [112]. Leptin stimulates LH secretion, as ascertained by correction of LH pulses following leptin administration in women with fasting-induced HHOA dysfunction [113]. Because hypothalamic GnRH-secreting cells do not express leptin receptors, stimulation of LH secretion appears to be an indirect effect, possibly through AgRP/NPY and POMC neurons, which do express the receptor and are anatomically associated with GnRH neurons [114]. Another pathway for leptin-induced GnRH secretion involves kisspeptin; a neuropeptide expressed in the arcuate and periventricular nuclei, which binds to its receptor in hypothalamic GnRH-expressing cells, inducing its secretion [115]. Leptin also exerts direct effects in all ovarian cells and seems to have a physiologic regulatory effect in folliculogenesis [111].

In the context of PCOS, the role of leptin has been subject to profound controversy, with opposing views regarding its true participation. Because leptin concentrations are consistently found to be strongly correlated with weight, some reports consider the hyperleptinemia seen in PCOS as only a byproduct of this condition [116]. On the other hand, findings linking leptin levels to estradiol [117], testosterone, and insulin [118] in women with PCOS advocate for a more complex role of leptin in its pathophysiology. Moreover, reports of elevated leptin in nonobese PCOS patients further question quantitative adiposity as the sole origin of hyperleptinemia in this scenario [119].

Regardless of its origin, in PCOS hyperleptinemia exerts direct effects on ovarian physiology by arresting follicle development [111]. Moreover, ovarian paracrine and autocrine leptin signaling may also be disrupted, parallel to alterations in the HHOA [120]. Likewise, *in vitro *studies show a decrease in collagenase expression in ovarian tissue after exposure to high concentrations of leptin, adding to the ovulatory disturbances in this scenario [121].

In obese women with PCOS, the consequences of hyperleptinemia may be excacerbated by lower levels of sOB-R, which increase leptin ligand availability. Indeed, sOB-R expression appears to be inverse to both adiposity and DHEAS levels [122]. Finally, matters may be further complicated with the development of leptin resistance, a state of diminished response to the neuroendocrine effects of this hormone, namely, regarding attenuation of appetite [123]. Several factors may contribute to this phenomenon, including saturation of blood brain barrier transporters of leptin, saturation and downregulation of its receptors in kisspeptin-expressing neurons, obesity-induced endoplasmic reticulum stress in target neurons, and a negative feedback loop engrained within its own signaling cascade, which activates in the face of leptin overactivity [124]. In PCOS, leptin resistance results not only in insufficient permissive signaling to achieve optimal LH pulse secretion [125], but also in defective suppression of appetite, which may perpetuate obesity [126].

## 8. Polycystic Ovary Syndrome in Nonobese Women: Key Differential Features

Given that obesity is only present in roughly half of all cases of PCOS, efforts have been directed to the description of pathophysiologic mechanisms in nonobese women. In this aspect, differential patterns of insulin physiology are paramount in characterizing the metabolic profiles of obese and nonobese women with PCOS [127]. Although nonobese women exhibit lower insulinemia and IR [128] associated with higher SHBG levels [129], hyperinsulinemia is still a common finding in this population [127]. Indeed, several studies have suggested a primary alteration in *β* cell function as a pathophysiological component independent of weight [130, 131]. Insulin hypersecretion has been suggested as the probable underlying mechanism in this scenario, in light of reports underpinning increased *β* cell activity—and not IR—as a predictor of hyperandrogenemia in PCOS women [132], being present in both lean and obese subjects [133]. This intrinsic disruption in *β* cell function may be linked to *in utero* exposure to elevated androgen levels [134], as supported by animal models wherein this kind of fetal stimulation resulted in altered expression of genes associated with *β* cell function and mass as well as altered insulin secretion response *in vitro* and altered *β* cell quantity [135]. Nevertheless, despite strong evidence for candidacy, variations of the insulin gene VNTR (variable number tandem repeat) minisatellite have been demonstrated not to play a relevant role in the development of PCOS [136], raising the need for further research on the subject.

Another element contributing to hyperinsulinemia in this scenario is increased endogenous opioid signaling by virtue of elevated levels of *β* endorphins in peripheral circulation. These messengers favor the development of hyperinsulinemia by stimulating its pancreatic secretion and inhibiting its hepatic clearance, as well as regulating IGFBP-I serum levels, therefore modulating IGF-I bioavailability, although the origin of these greater levels of endogenous opioids remains unclear [137].

Nonobese individuals also show greater LH/FSH ratios, which seem to play a major role in this subset of subjects [138]. In this scenario, endogenous opioids also appear to play an important role, albeit in this case, within the central nervous system rather than peripherally. Indeed, an increased opioid signaling tone is observed during the late follicular and luteal phases, associated with slower LH pulse secretion, in physiologic conditions [139]. However, a decreased opioid tone is observed in PCOS, accompanied by increased sensitivity to GnRH, resulting in reinforced LH secretion [137].

Lean women with PCOS have been found to display diminished sensitivity to catecholamine-mediated lipolysis in SAT [140], resulting in preservation of this tissue. Because leptin is primarily secreted from SAT [108], this may partially explain hyperleptinemia found in normal-weight women with PCOS, and although intrinsic dysregulation of its secretion mechanisms may also be involved, the precise chain of events remains unelucidated [119].

Likewise, while obese women with PCOS show higher levels of testosterone than adrenal androgens, this relationship is inversed in nonobese subjects, suggesting that adrenal steroidogenesis may be more severely altered in lean individuals [17]. In addition, although both obese and nonobese women with PCOS have increased adrenal sensitivity to ACTH for cortisol and androgen secretion [141], the increased ratio of the 11-oxo metabolites of cortisol and corticosterone to their 11-hydroxy metabolites in urine of nonobese PCOS women indicates enhanced oxidation by 11-*β*–hydroxysteroid dehydrogenase (11-*β*-HSD) in these subjects. In turn, this effect would result in increased cortisol clearance, allowing for greater adrenal androgen synthesis. This 11-*β*-HSD upregulation has been proposed to be due to hyperandrogenemia or hyperinsulinemia, although further research is required [142].

Lastly, oxidative stress and low-grade inflammation are two features equally shared by obese and nonobese PCOS subjects [143]. Because the genesis of this component does not seem to be attributable to body composition in nonobese women with PCOS, leading premises are genetic in nature, with the proposal of overexpression of several proinflammatory genes as the cause underlying this phenomenon in this subset of females [144].

## 9. Chronic Inflammation: From Insulin Resistance to Polycystic Ovaries and Beyond

Recent evidence describes a central role for certain proinflammatory mediators in the pathophysiology of PCOS, posing a new focus on the etiological considerations for PCOS, which is currently considered a chronic, low-grade inflammatory disorder, independently of the presence of obesity [145], although these phenomena are indeed exacerbated by adiposity [146]. Reports show that women with PCOS, both with obesity and normal weight, exhibit elevated serum TNF, C-reactive protein (CRP), monocyte and lymphocyte circulating levels, and inflammatory infiltration in ovarian tissue [147].

Several mechanisms have been proposed to explain these features. Among these, the existence of proinflammatory genotypes has been suggested, including polymorphisms of sequences codifying TNF, TNF receptor, IL-6, and IL-10 [148]. Remarkably, greater expression of the CD11c gene is associated with greater proinflammatory macrophage infiltration is SAT and VAT, favoring a transition to decreased secretion of adiponectin and increased TNF and leptin secretion from adipocytes [149].

The role of TNF is especially important in the setting of IR and PCOS. In addition to the diminishing effect it bears over insulin sensitivity, through serine phosphorylation of IRS-1 by PKC [16]. This cytokine also stimulates steroidogenesis and proliferation in TC, as well as follicular atresia, contributing to hyperandrogenemia, posing yet another level of complexity and self-perpetuation for PCOS [150].

Furthermore, hyperglycemia may contribute to inflammation in PCOS, possibly explaining the greater magnitude of manifestations in PCOS (Figure 3). Circulating mononuclear cells utilize glucose as their main redox substrate, with part of its metabolites going into the pentose-phosphate pathway to yield NADPH [151]. Oxidation of this molecule leads to the production of reactive oxygen species (ROS), which induce oxidative stress, with the subsequent activation of NF-kB, a transcription factor involved in the expression of proinflammatory mediators such as TNF and IL-6 [66]. Hence, hyperglycemia may result in increased ROS production. Additionally, oxidative stress appears to also induce key steroidogenic molecules in TC, namely, CYP11A1, CYP17A1, 3-*β*-HSD, and StAR, favoring hyperandrogenemia [152].

Additionally, hyperleptinemia boosts its effects in immune function regulation, including production of proinflammatory cytokines, such as TNF, IL-6, and IL-12, stimulation of polymorphonuclear cell chemotaxis, inhibition of lymphocyte apoptosis by suppression of Fas-mediated signaling, and induction of Th1 differentiation, both in obese and nonobese females with PCOS [153].

Beyond these implications in PCOS, the reinforcing cross-talk between IR and chronic inflammation generates a welcoming environment for the development of further cardiometabolic disturbances. Oxidative stress caused by ROS production in immune cells plays a fundamental role in the genesis and progression of endothelial dysfunction, which leads to the development of arterial hypertension, and cardiovascular disease [154]. Furthermore, chronic inflammation and IR are two essential elements in the etiopathogenesis of MS and DM2, which in turn open the door for further complications for the overall health of women with PCOS [155].

## 10. Concluding Remarks

Given the pivotal role IR and obesity play in the etiopathogenesis and progression of PCOS and its potential subsequent metabolic and cardiovascular complications, both should be considered essential therapeutic targets [156]. Although traditionally metformin is thought of as a hallmark of PCOS treatment as the mainstay insulin sensitizer, the advent of the distinct phenotypes for this syndrome and the broader acceptance of this categorization bring into question its indication in all cases of PCOS [157]. Uncertainties are even more commonplace surrounding other pharmacologic alternatives habitually considered for PCOS management, such as thiazolidinediones and statins [158, 159].

Lifestyle modifications in the treatment of PCOS do not escape criticism and controversy despite being widely accepted recommendations. Physical activity (PA) has been reported to ameliorate anovulation, IR, blood pressure, and lipid profiles in women with PCOS, sometimes independently of weight loss [160]; yet PA alone does not seem to be able to equal these parameters to non-PCOS subjects [161]. Therefore, it should be accompanied by a complementary diet plan in order to fully potentiate the effects of a lifestyle-modification therapeutic program. Standard weight loss programs may be sufficient for ameliorating features of PCOS, in the form of a nutritionally adequate, fiber-rich, low fat, moderate protein, and high carbohydrate intake diet with a 500–1,000 kcal/day reduction [162]. On the other hand, more specialized low-carbohydrate ketogenic diets have been reported to significantly reduce weight, LH/FSH ratio, testosterone and fasting insulin ratio, and IR in women with PCOS [163], although concerns on their safety on lipid profiles [164] and cardiovascular risk [165] call for careful consideration. Ultimately, a wide range of dietary programs akin to these general concepts are accepted to similarly improve weight, reproductive and metabolic variables in PCOS so long as they boast nutritional adequacy and long term sustainability [166]. In addition, novel perspectives into the pathophysiology of PCOS, such as the role of ovarian AGE deposition, suggest avoidance of glycotoxin-rich diets as an added recommendation [167].

In light of the controversial considerations regarding treatment of this syndrome, further studies are required to elucidate the intricacies within the pathophysiology of PCOS and the true relationships between IR, hyperinsulinemia, and hyperandrogenemia, as well as other important hormonal disturbances, particularly alterations within steroid hormone metabolism, hyperleptinemia, and leptin resistance. This would allow for the formulation of improved approaches to the management of obesity and IR as therapeutic targets in women with PCOS, in order to improve their quality of life and especially their fertility outcomes, which are often the main concern of both patients and physicians in the management of this pathology.

## Figures and Tables

**(a):** 

Manifestation	Reference	Sample	Prevalence
Manifestations of hyperandrogenism			

Hirsutism	Marcondes et al. [34]	73	83.8%
	Baldani et al. [35]	365	73.2%
	Jedel et al. [36]	30	73%

Acne	Jedel et al. [36]	30	63%
	Özdemir et al. [37]	115	53%
	Baldani et al. [35]	365	49.6%

Alopecia	Özdemir et al. [37]	115	34.8%
	Sivayoganathan et al. [38]	70	16%

Seborrhea	Özdemir et al. [37]	115	34.8%

Manifestations of ovarian dysfunction			

Oligomenorrhea	Valkenburg et al. [39]	412	74%
	Baldani et al. [35]	365	69.2%
	Jedel et al. [36]	30	20%

Amenorrhea	Valkenburg et al. [39]	412	26%
	Baldani et al. [35]	365	21.5%
	Jedel et al. [36]	30	43%

Ultrasound polycystic ovaries	Baldani et al. [35]	365	97.3%
	Valkenburg et al. [39]	412	89%
	Hahn et al. [40]	200	83%

**(b):** 

Condition	Reference	Sample	Prevalence
Associated conditions			

Obesity	Ehrmann et al. [41]	394	80%
	Hahn et al. [40]	200	52%
	Azziz et al. [42]	267	42%

Insulin resistance	Carmina and Lobo [43]	267	77%
	Hahn et al. [40]	200	71%

Impaired fasting glucose	Ehrmann et al. [41]	122	35%
	Legro et al. [44]	254	31.1%

Type 2 diabetes mellitus	Legro et al. [44]	254	7.5%
	Ehrmann et al. [41]	394	6.6%

Arterial hypertension	Ehrmann et al. [41]	394	21%
	Elting et al. [45]	346	9%

Dyslipidemia	Hahn et al. [40]	200	46.3%
	Ehrmann et al. [41]	394	32%

Metabolic syndrome	Dokras et al. [46]	129	47.3%
	Apridonidze et al. [13]	106	43%

Mood disorders	Jedel et al. [36]	30	53%
	Hollinrake et al. [47]	103	21%

All studies cited were carried out in adults.

**Table 2:** Diagnostic criteria for polycystic ovary syndrome.

	Clinical or biochemical hyperandrogenism	Oligo/anovulation	US finding of polycystic ovaries∗
NIH, 1990 Both of the following:	+	+	

ESHRE/ASRM, 2003 Only 2 of the following:	+	+	+

AES, 2006 All 3 of the following:	+	+	+

NIH: National Institute of Health of the United States; ESHRE: European Society of Human Reproduction and Embryology; ASRM: American Society of Reproductive Medicine; AES: Androgen Excess and PCOS Society.

All sets of criteria require the exclusion of other etiologies such as congenital adrenal hyperplasia, androgen-secreting neoplasms, and Cushing's síndrome.

*Ultrasound polycystic ovaries can be defined as the presence of ≥12 follicles of 2–9 mm width or an increase in ovarian volume (>10 mL) in at least one ovary, in women not consuming oral contraceptives.

## Abbreviations

11-*β*-HSD: 11-*β*-hydroxysteroid dehydrogenase
3-*β*-HSD: 3-*β*-hydroxysteroid dehydrogenase
ACTH: Adrenocorticotropic hormone
AGE: Advanced glycation end products
AgRP: Agouti-related protein
AMH: Anti-Müllerian hormone
cAMP: Cyclic adenosine monophosphate
CRP: C-reactive protein
CRH: Corticotropin-releasing hormone
CYP11A1: Cholesterol side-chain cleavage enzyme
CYP17A1: 17-*α*-Hydroxylase/17,20-lyase
CYP19A1: Aromatase
DHEAS: Dehydroepiandrosterone sulfate
DHT: Dihydrotestosterone
DM2: Type 2 diabetes mellitus
FFA: Free fatty acids
FSH: Follicle-stimulating hormone
GC: Granulosa cells
GLUT4: Glucose transporter type 4
GnRH: Gonadotropin-releasing hormone
GSK-3: Glycogen synthase kinase 3
HHAA: Hypothalamus-hypophysis-adrenal axis
HHOA: Hypothalamus-hypophysis-ovary axis
HNF4*α*: Hepatocyte nuclear factor 4-*α*
IGF-1: Insulin-like growth factor-1
IGFBP-1: Insulin-like growth factor-1 binding protein
INSR: Insulin receptor
IR: Insulin resistance
LDL-C: Low-density lipoprotein cholesterol
LH: Luteinizing hormone
MAPK: Mitogen-activated protein kinase
MS: Metabolic syndrome
mTOR: Mammalian target of rapamycin
mRNA: Messenger ribonucleic acid
NADPH: Nicotinamide adenine dinucleotide phosphate
NPY: Neuropeptide Y
OB-R: Leptin receptor
PA: Physical activity
PCOS: Polycystic ovary syndrome
PI3K: Phosphoinositide 3-kinase
PKA: Protein kinase A
PKC: Protein kinase C
POMC: Proopiomelanocortin
ROS: Reactive oxygen species
SAT: Subcutaneous adipose tissue
SHBG: Sex hormone binding globulin
sOB-R: Soluble leptin receptor
StAR: Acute steroidogenic regulatory protein
TC: Theca cells
TGF-*β*: Transforming growth factor *β*
TIRE: Thymine-rich insulin response elements
TNF: Tumor necrosis factor
VAT: Visceral adipose tissue.
